# The Organismal Form and Function Lab-Course: A New CURE for a Lack of Authentic Research Experiences in Organismal Biology

**DOI:** 10.1093/iob/obz021

**Published:** 2019-08-23

**Authors:** C E Oufiero

**Affiliations:** Department of Biological Sciences, Towson University, Towson, MD 21252, USA

## Abstract

There are many benefits to engaging students in authentic research experiences instead of traditional style lectures and “cookbook” labs. Many Course-based Undergraduate Research Experiences (CUREs) have been developed that provide research experiences to a more inclusive and diverse student body, allow more students to obtain research experiences, and expose students to the scientific process. Most CUREs in the biological sciences focus on cellular and molecular biology, with few being developed in ecology, evolution, and organismal biology. Here, I present a one-semester CURE focused on organismal form and function. The goal of the course was to have students develop their own research questions and hypotheses in relation to invertebrate form and movement, using high-speed cinematography to collect their data. In this paper, I describe the motivation for the course, provide the details of teaching the course, including rubrics for several assignments, the outcomes of the course, caveats, and ways a similar course can be implemented at other institutions. The course was structured to use a scaffolding approach during the first half of the semester to provide the content of form–function relationships and allow students to acquire the laboratory skills to quantify animal movement. The second half of the course focused on student-driven inquiry, with class time dedicated to conducting research. As there is a push to engage more students in research, I hope this course will inspire others to implement similar classes at other universities, providing a network of collaboration on integrative organismal student-driven research.

## Synopsis

O Curso Prático Sobre Forma e Função Organismal: um Novo CURE Para a Falta de Experiências de Pesquisa Autênticas em Biologia Organismal (The Organismal Form and Function Lab-Course: a New CURE for a Lack of Authentic Research Experiences in Organismal Biology) Há muitos benefícios em envolver os alunos em experiências de pesquisa autênticas, em vez de aulas práticas no estilo tradicional de palestras e de “livros de receitas.” Muitas Experiências de Pesquisa de Graduação baseada em Cursos (CURE, em inglês) foram desenvolvidas para fornecer experiências de pesquisa a um corpo discente mais inclusivo e diversificado, permitindo que mais alunos obtenham experiências de pesquisa e os exponham ao processo científico. A maioria das CUREs nas ciências biológicas se concentra na biologia celular e molecular, com poucas sendo desenvolvidas em ecologia, evolução e biologia organismal. Aqui, apresento um CURE de um semestre focado na forma e função organismal. O objetivo do curso era fazer com que os alunos desenvolvessem suas próprias questões e hipóteses de pesquisa em relação à forma e ao movimento dos invertebrados, usando a cinematografia de alta velocidade para coletar seus dados. Neste artigo, descrevo a motivação para o curso, forneço os detalhes do ensino, incluindo pontos para várias tarefas, os resultados do curso, ressalvas e maneiras pelas quais um curso semelhante pode ser implementado em outras instituições. O curso foi estruturado de maneira a usar uma abordagem construtiva durante a primeira metade do semestre, fornecendo o conteúdo das relações de função de forma e permitindo que os alunos adquirissem as habilidades de laboratório para quantificar o movimento dos animais. A segunda metade do curso concentrou-se na investigação centrada no aluno, com o tempo de aula dedicado à realização de pesquisas. Como há um esforço para engajar mais estudantes na pesquisa, espero que este curso inspire outros a implementar aulas semelhantes em outras universidades, proporcionando uma rede de colaboração em pesquisas integrativas organizadas por estudantes. Translated to Portuguese by G. Sobral (gabisobral@gmail.com)

## Introduction

### What is a CURE and what types have been developed?

It is well established that engaging students in research experiences is beneficial in terms of student’s understanding what it’s like to think like a scientist, improving student’s ability to analyze data, increasing graduation rates in STEM, and making research experiences more inclusive (Bangera and Brownell 2014; Brownell et al. 2015; Rodenbusch et al. 2016), which has led to the development of entire Course-based Undergraduate Research Experiences (CUREs; Table 1). Unlike traditional laboratory-based courses, CUREs lack traditional “cookbook” labs, where the outcomes are known to the instructor. Instead, CUREs focus on student driven inquiry and active participation in the scientific process of experiments where the outcomes are often unknown to the instructor (Auchincloss et al. 2014; Dolan 2016). Within the life sciences, there are an increasing number of CUREs being offered at institutions (Dolan 2016; Table 1) that span the range of activities and levels of engagement of students in the scientific process. These courses benefit both the instructors and the students. The benefits to the students include research experiences, increased retention in STEM related disciplines, and ownership of the research project. Benefits to the instructor include integrating research with teaching, which is a benefit in primarily teaching based institutions, long-term collection of large datasets, and training more students to participate in independent research. CUREs are also beneficial in that they offer authentic research experiences to a larger diversity of students, who may not be able to participate in independent research within a research lab due to limited availability in research labs and limited time available from students that need to balance course-work and jobs (Auchincloss et al. 2014). Given all of the benefits provide by CUREs, it is no surprise more are being offered. However, most of these courses are primarily cell and molecular biology focused, often instructing students in laboratory techniques, such as PCR (Table 1). Several have begun to be developed that introduce students to ecology, often with a lab component such as microbiome analyses (Fukami 2013). Rarely are CUREs offered that instruct students in research experiences in integrative organismal biology (Full et al. 2015). In this paper, I describe a one-semester CURE focused on organismal form and function, providing the course outline and schedule, example assignments and rubrics, and equipment used; outcomes of the first iteration of the course; challenges associated with teaching such a course; and provide suggestions for implementation of a similar course at other institutions.

**Table 1:** Course-based Undergraduate Research Experiences at other institutions

Source from Dolan (2016)	Topic	Journal
Alkaher and Dolan (2014)	Intro bio lecture, upper level plant biology	Book chapter
Bascom-Slack et al. (2012)	Plant microbiome	Science
Boltax et al. (2015)	Cell molecular and organic chemistry	Biochemistry and Molecular Biology Education
Brownell et al. (2015)	Point mutation in p53 gene	CBE-Life Sciences Education
Burnette and Wessler (2013)	Eukaryotic transposable elements	Genetics
Chen et al. (2005)	Functional Genomics	PLOS Biology
Drew and Triplett (2008)	Genome sequencing	Journal of Microbiology and Biology Education
**Fukami (2013)**	**Plant pollinator microbiome interactions**	**Science**
**Full et al. (2015)**	**Organismal**	**Integrative and Comparative Biology**
Harrison et al. (2011)	Phage genomics	CBE-Life Sciences Education
Harvey et al. (2014)	Gene expression	CBE-Life Sciences Education
Hatfull et al. (2006)	Phage genomics	PLoS Genetics
Jordan et al. (2014)	Phage genomics and discovery	mBio
Kloser et al. (2011)	Ecology	PLoS Biology
Kloser et al. (2013)	Ecology	Journal of College Science Teaching
Makarevitch et al. (2015)	Gene expression	CBE-Life Sciences Education
Rowland et al. (2012)	Biochemistry	Biochemistry and Molecular Biology Education
**Russell et al. (2015)**	**Biodiversity, genetics, molecular identification of insects**	**CBE-Life Sciences Education**
Shaffer et al. (2010)	Genomics/Bioinformatics	CBE-Life Sciences Education
Shaffer et al. (2014)	Genomics/Bioinformatics	CBE-Life Sciences Education
Shanle et al. (2016)	p300 Bromodomain mutations	Biochemistry and Molecular Biology Education
Shapiro et al. (2015)	Most CMB, one ecology plant-microbiome	Journal of Microbiology & Biology Education
Simmons (2014)	CMB	FASEB Journal
Siritunga et al. (2011)	Genetics	CBE-Life Sciences Education
**Ward et al. (2014)**	**Botany, some organismal/physiology focus**	**CBE-Life Sciences Education**
Wiley and Stover (2014)	Genetics	CBE-Life Sciences Education
Wolkow et al. (2014)	Yeast genetics	CBE-Life Sciences Education

Citations in bold represent other organismal focused CUREs.

### The organismal form and function lab

In conjunction with the Towson University Research Enhancement Program, which was funded by a Howard Hughes Medical Institute Inclusive Excellence Initiative, I took the opportunity to develop an integrative organismal based CURE drawing inspiration from marine lab-based courses, laboratory exercises in animal physiology, and my own research (e.g., Oufiero et al. 2016). The research in my lab is diverse and has spanned studies of growth and development in lizards (Oufiero and Angilletta 2006), to variation in swimming performance in fish (Oufiero et al. 2011, 2014), feeding in vertebrates and invertebrates (Oufiero et al. 2012, 2016), and has included collaboration on a variety of organisms (Van Sant et al. 2012; Schmitz et al. 2013). One of the consistent techniques I have used throughout a majority of my research is high-speed cinematography to characterize the movement of animals (e.g., performance and kinematics of swimming and feeding). I have also had undergraduate students collaborate on many aspects of these projects, instructing them on how to film animals, how to digitize the videos to obtain the behavior or performance of interest, and how we get a set of kinematic and performance variables from the digitized points. Like professors at many universities, I do not receive course buy out by taking undergraduate students in my lab to conduct independent research (our BIOL 490 and 491), so there is a limit to the number of students I can have in my personal research lab. This creates a bottleneck for the number of students I can engage in research. However, as I worked with more students, and on more diverse organisms, I began developing protocols and exercises to instruct students on how to film, digitize, and obtain kinematics. I introduced a 1-week recitation assignment in animal physiology (BIOL 325) where students used high-speed videos my research lab obtained on praying mantis feeding to develop their own questions about feeding performance. Using ImageJ (https://imagej.nih.gov/ij/) and videos of praying mantis feeding (e.g., Oufiero et al. 2016), students collected data such as predator–prey distance, speed of the strikes, and length of the foreleg. Similar exercises have since been applied in the Phylogenetic Analysis of Vertebrate Structure course (EVE 105) at the University of California Davis (S. J. Longo, personal communication). These experiences partly inspired the development of the organismal form and function CURE.

I modeled this course based on my own experience in a similar course, the Fish Swimming Course offered through the University of Washington’s Friday Harbor Marine Labs (https://fhl.uw.edu/courses/course-descriptions/course/fish-swimming-kinematics-ecomorphology-behavior-and-environmental-physiology-2019/). This is a 5-week course focused on aspects of fish swimming, such as ecomorphology, taught by Drs. Paolo Domenici, John Steffensen, and Guy Claireaux (summer 2005). The structure of the course included traditional style lectures the first week and a half of class, which teaches the principles of fish swimming in ecological and biomechanical contexts. It included several collecting trips to obtain specimens to work on throughout the remainder of the course, group work, and hypothesis testing. The majority of time in the course was dedicated to collect, analyze, and present data based on our hypothesis. This was a great experience for many reasons, including research experience, hypothesis testing, and group work with students from all over the world. However, the Fish Swimming course, and similar courses at other institutions, are limited in the students that can attend and costs additional money (although there are sources for students to obtain funding: https://fhl.uw.edu/courses/fellowships-scholarships/). My goal was to offer research experiences in a one semester CURE that any university could offer by combining my personal research with the course layout of summer research courses such as the Fish Swimming course offered at Friday Harbor Labs.

## Materials and methods

### Development of the organismal form and function CURE at Towson

The aim for the development of the organismal form and function CURE was to provide more students the opportunity to conduct research. The overall goal of the course was for students to develop and test their own research questions in relation to animal movement using high-speed videos. Using the quantification of animal movement with high-speed videos the objectives for the course were for students to:

1. gain experience in organismal biology with a focus on form, function, and performance relationships;
2. obtain a set of hard (e.g., data collection and analysis) and soft (e.g., time management and group work) skills;
3. develop and test their own hypothesis/question;
4. present their research to a scientific and general audience;
5. gain ownership of their research and data.

Because of the complex, integrative nature of animal movement, the relative ease of collecting data on animal movement with high-speed cameras, and the potential for new discoveries, I chose to focus on organismal form and function. This approach provided an overarching theme for the course, but allowed for the flexibility for student creativity in developing their question. Many CUREs are based in cell and molecular biology and focus on the instructor’s research, so questions may be limited (Table 1). I wanted to allow room for creative thinking and unique research questions. While it is nearly impossible to instruct students in a course that is completely open in terms of the research questions, using the broad umbrella of organismal form and function kept the questions focused but flexible.

I took several approaches to provide a unique research opportunity with open ended questions in a course where you still need to provide a letter grade to the students and a framework so it remained organized throughout the semester. First, I decided on a framework with some lectures and paper discussions at the beginning to give students some basic content, but the students were required to develop the research questions to test throughout the course. The scaffolding approach intends to build upon knowledge, giving students new pieces each week (Johnson and Smith 2008; Alkaher and Dolan 2016). Having traditional lectures at the beginning of the course was intended to provide the basic content for students to begin asking questions about form/function relationships. I also included paper discussions and book chapters on several of the topics so students could obtain an idea of the types of questions that scientific papers address (Table 2).

**Table 2:** List of topics covered and the associated readings during the scaffolding phase of the course

Week	Topic	Readings
1	Form, function, and performance	Irschick and Higham (2015, Chap. 1)
2	What affects performance?	Irschick and Higham (2015, Chap. 1) Winchell et al. (2018)
3	Muscle physiology and energetics	Biewener and Patek (2018, Chap. 2)
4	Terrestrial locomotion	Full and Tullis (1990) Biewener and Patek (2018, Chap. 4)
5	Jumping, climbing, and clinging	Burrows and Sutton (2008) Biewener and Patek (2018, Chap. 7)
6	Flying	Crall et al. (2015) Biewener and Patek (2018, Chap. 6)

Second, I created assignments that focused on developing the students as researchers in animal movement. For example, some of the most important aspects of high-speed videos are the videos themselves. I simply turned what I look for in a video to analyze movement into an assignment (Supplementary Material). The rubric grades the student videos based on its quality for digitizing and obtaining kinematics. The rubric includes whether the video is in focus, there is enough lighting, it captures the behavior of interest, and if the frame rate and shutter speed are adequate to minimize blur.

Third, I kept the development of research questions a constant theme through planned and unplanned activities throughout the first 7 weeks of the course. To facilitate the development of research questions I used several approaches. First, starting early in the semester (week 2), I had a small assignment where the students turned in two observations from nature on factors that may affect animal performance (e.g., one student submitted the observation that insects with different sized wings might perform differently in wind). Second, I decided to have paper discussions that focused on some potential questions (Table 2), such as the effects of urbanization on form–function relationships (Winchell et al. 2018), running on different inclined surfaces (Full et al. 1990), effects of hindlimb length on jumping (Burrows and Sutton 2008), and how obstacles effect flight performance (Crall et al. 2015). The intent was to show students how they could start addressing questions of form, function, and performance relationships. Third, I obtained some invertebrate animals from Carolina Biological to ensure there was a live animal to film at the start, as getting an animal to perform can be difficult. Lastly, I continued discussions of potential projects throughout the early weeks, through both planned exercises including additional assignments of potential research questions, and unplanned discussions. Later, after each individual student submitted their formal research question, during one of the field trips to collect, I had them pair up and change partners to discuss their initial hypotheses they turned in for an assignment. This allowed me to determine which students were interested in which organisms and questions. We also spent some time in class writing out potential research questions on the board, discussing sample sizes and experimental groups to test the question.

Fourth, after the scaffolding approach and groups were formed, I treated the class as lab time using directed student inquiry (Germann 1989, 1991; Brickman et al. 2009). The class was designed to be similar to conducting research in a research lab, only the students had dedicated course time to work on their projects with me, an undergraduate learning assistant (ULA), and grad student there to help, while the students received course credit. Course time was also dedicated to more traditional “lab meeting” time as is often done in research labs, during this time I met with students in class to discuss their projects and we had time for student presentations of primary literature. It is often difficult to find time for research lab meetings based on many people’s schedules, therefore an added benefit of teaching a CURE was having dedicated time to meet with students and discuss research.

Finally, I switched to an invertebrate system. While I had only begun exploring performance of invertebrates with praying mantids (Oufiero et al. 2016), I decided they would be a better model for the course for several reasons. First since invertebrates are not covered under current Institutional Animal Care and Use Committees (IACUC) we would not have to spend time writing protocols, training, or be confined in the animals or questions students could ask. However, I did give basic training on the ethics of working with live animals during the first lecture. Second, there is a greater diversity of invertebrates in Maryland (∼500 arthropods alone), providing ample opportunity for research questions. As I am not an expert on all invertebrates, the students were able to take ownership of their research by teaching me about the natural history and general biology of the organisms they chose to work on. Furthermore, offering the course in the fall, the goal was to ensure we would have a better chance at collecting a greater diversity as many arthropods will be in the adult life stages. And lastly, while there has been a lot of research on performance of vertebrate models, there is much less on the diversity of invertebrates, again offering lots of opportunities for unique student driven questions. One aspect of teaching a CURE is discovery, switching to an invertebrate model provided the potential for students to make new discoveries, helping to highlight basic scientific research in the context of hypothesis testing. Furthermore, the form, function, and performance model in invertebrates may also provide the opportunity for students to publish their results if they make new discoveries (e.g., Davis et al. 2019). Providing the opportunity for students to formulate a hypothesis, design their experiment, collect and analyze data, and potentially publish or present their results to the broader scientific community is another aspect that sets CUREs apart from traditional cookbook based labs (Auchincloss et al. 2014).

Using a model that is not the focus of my direct research was also a benefit. While many CUREs are developed in direct conjunction with an instructor’s research, this may add additional stress if projects are not going as planned. That is, if an instructor is relying on the data collected in the CURE for their personal research there may be less student driven inquiry and more input from the instructor on the types of questions that can be addressed. Focusing on the use of high-speed videos to quantify animal movement on any potential invertebrate removed the stress of me having to collect data. If the students’ projects failed, it would not be a detriment to my research program, and the students still gain authentic research experience. If the students succeed or make a new discovery they could continue investigating the question through independent research credit. It may therefore be beneficial to focus on a subject that is more tangentially related to the professor’s research program.

### Application of the organismal form and function CURE

**Fig. 1:**
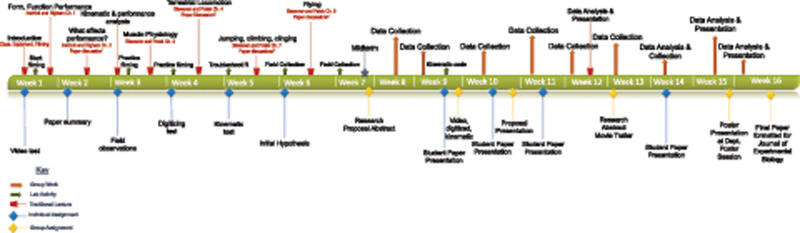
Timeline of course activities. Top of the timeline represents activities in the course, with traditional lectures presented the first 7 weeks of the course using a scaffolding approach, and student directed inquiry the second 7 weeks. Bottom portion of the timeline represents assignments throughout the semester, starting with individual assignments (blue diamonds) to assess students on techniques to quantify animal movement with high-speed cameras; followed by more group assignments (yellow diamonds) to assess students on presenting their research.

Under a 16-week semester, I split the course into scaffolding and student driven inquiry with a midterm at week 7 on basic content. The class consisted of a 4-h period on Mondays, similar to a traditional “lab” time and a 2-h period on Wednesdays similar to a traditional “lecture” time. Figure 1 outlines the timeline of the course, including lectures, readings, and both individual and group assignments (see also Supplementary Material). The goal during the scaffolding section of the course was to provide students the basic content of animal movement, focused on the physiology and biomechanics in an ecological and evolutionary context. Providing the first two chapters of Irschick and Higham’s Animal Athletes book (2015) at the start of the course gave the context of what animal form, function, and performance are and how they are related to ecology and evolution. During the “lab” portion of this section of the course the students were trained and evaluated (Supplementary Material) on using the high-speed cameras and associated camera software to capture movement, including an initial video assignment filming anything (e.g., fingers snapping and coin dropping). Students were also required to find and write a short summary of an article from the primary literature on animal movement, introducing them to researching the primary literature related to their topic early.

The remaining time during the scaffolding section of the course included lectures on some of the more common animal locomotor modes, learning to digitize and obtain kinematics, and begin to formulate questions. During this portion of the course, I used several chapters from the recent edition of Animal Locomotion (Biewener and Patek 2018), focusing on muscle physiology, terrestrial locomotion, jumping, and flying as these seemed to be locomotor modes we might encounter in invertebrate models. The goal was to provide content on how animals actually move. During each lecture there was also a paper from the primary literature on the particular form of locomotion and how it might vary in relation to morphology and/or ecology (Table 2). The goal at week 7, midway through, was to have animals collected, hypotheses formulated, and basic content covered (Fig. 1). To assess students on basic content a short-answer midterm was used. Using the hypothetical Rhinogradentia creatures (Gonzalez-Voyer and Von Hardenberg 2014) students were asked questions about form, function, and performance in relation to the content we had covered. The “lab” portion of the course during this time was dedicated to practice filming, having all the students gain experience with the different cameras that were available, digitizing using the MtrackJ plugin (https://imagescience.org/meijering/software/mtrackj/) for ImageJ, and learning basic kinematics (displacement, velocity, and acceleration) using R (R Core Team 2014). Instructing on the use of the high-speed cameras initially, with some lab crayfish and cockroaches provided them a context for what could potentially be asked. To obtain specimens to work with, give students some field collecting experience, and provide additional motivation for questions, field trips were planned during our Monday class, two to Oregon Ridge Park (https://www.oregonridgenaturecenter.org) and one to the Towson University Field Station. This is similar to the collecting trips in the FHL Fish Swimming course.

The second half of the course shifted to student driven inquiry, where most of the class time was dedicated to data collection and analyses, as well as student presentations on their research proposal and primary literature. The goal for presentation on their research proposal was to establish their experimental design, the filming protocol, sample sizes, what point they were tracking, etc., to ensure they knew what videos to obtain to address their questions. This is similar to what many research labs do in lab meetings. Similarly, the individual student presentations on the primary literature ensured students were looking to the primary literature to develop their research questions. During this student-driven inquiry portion of the course the ULA, grad student, and myself were available to support the students in their research. Initially, our role was to help determine a filming set up to get the performance data, and then shifted to data analysis. Students used kinematic code I developed that they had initially used, and expanded it where applicable, to get kinematic and performance metrics.

During the student driven inquiry portion of the class, I also assigned a unique assignment, where the students had to present their research abstract as a movie trailer. At the end of the semester each group had to present a poster of their research at the Department of Biological Sciences Fall Poster Day, as well as write a final paper on their project, formatted for the Journal of Experimental Biology. I had decided to incorporate social media in this class (including twitter @off_lab, https://www.youtube.com/channel/UCHwfwTS-hNyhZ9Xxi0BgVCA/featured, and a blog: wp.towson.edu/off-lab) as a means to highlight the research the students were conducting, share their videos of animal movement, integrate the importance of broader impacts, and allow the students to see the importance of communicating their research. The movie assignment was designed to incorporate creative elements in their research and elements of communicating their research. Students were provided with a rubric (Supplementary Material), which focused on content (e.g., was their hypothesis stated, were the methods mentioned, and was the organism mentioned), but also included a creative element (e.g., they could choose the genre of the movie).

## Results

### Outcomes of the first iteration of the organismal form and function CURE

The field trips were crucial to obtaining specimens and the development of research questions. While three trips were initially planned, weather is a factor that needs to be considered in future offerings of the course as our first three field trips were rained out (Supplementary Material). I had a contingency day planned, but we instead were only able to visit Oregon Ridge twice, which avoided the confounding factor of site of origin of research specimens on resulting performance. The first trip was essential for students to start formulating questions, for example I had not mentioned water striders, and once some students saw them they wanted to work with them.

A general challenge in teaching a CURE is getting students to develop their own, independent hypothesis in one semester that they can collect data on and test. Keeping the development of research questions a common theme throughout the first 7 weeks through each planned and unplanned exercise allowed students to hone in on specific research questions. After discussions of research projects, I had students form groups based on interests, as there was overlap in potential questions or organisms. This resulted in four groups with varying student composition (Table 3). Students then had to submit a group research abstract, so I could evaluate feasibility and help them refine their experimental approach, and ensure students would not get lost in designing their experiments. During our first data collection day, I met with each group independently to discuss filming setup, the performance variables they planned to obtain, how they were going to elicit the behavior from the animal, and data recording in excel. This was unplanned activity, but I realized was necessary, as some of the more menial tasks of science, are completely unknown at the start. Most groups focused on obtaining displacement, velocity, and acceleration of their animals, however, some groups separated their velocities to get vertical and horizontal, and some focused on getting heights. All groups used a 10th order polynomial to smooth the data (De Vries et al. 2012; Oufiero et al. 2016), which was discussed early for its importance in human digitized data to help remove error. During the first day of data collection the ULA and grad student were a big help to troubleshoot filming setups, they were also crucial for maintaining animals in the lab (e.g., did we need individual containers or were we keeping them in groups).

**Table 3:** Composition and project titles of the four groups during the first iteration of the organismal form and function CURE

Number of students	Gender ratio (F/M)	Project
2	1/1	Does body mass affect take-off velocity in brown marmorated stink bugs
3	3/0	Escape performance of crayfish with and without chelae
3	0/3	Effects of aquatic medium on water strider performance
4	2/2	The effects of hindlimb length and substrate on jumping performance of shortwinged grasshoppers

**Fig. 2:**
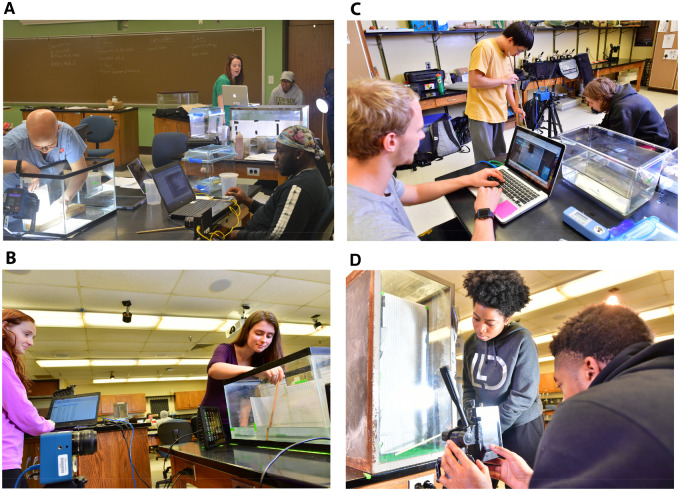
The four resulting groups and projects of the course. **A**) Team stinkbug collected videos on take-off flights of brown marmorated stink bugs (*Halyomorpha halys*), and examined the effect of body mass on take-off velocity. They also researched how to determine male and female and included sex in their statistical models. They used a Fastec IL3-S camera filming at 1000 Hz. **B**) Team crayfish examined the escape performance of *Cambarus acuminatus* crayfish with and without chelae, examining their height in the water column, velocity, and acceleration. They filmed at 500 Hz with an Edgertronic SC1 with IR filter removed, using 850 nm IR lights to illuminate the arena. **C**) Team water strider examined the effect of aquatic medium on the escape performance of water striders (*Aquarius remigis*), analyzing their distances traveled, velocity, and acceleration in one stride. They used an Edgertronic SC1 camera filming at 500 Hz, with overhead lights for illumination. **D**) Team jump compared jump kinematics, including height and vertical velocity, of short-winged grasshoppers (*Dichromorpha viridis*) on three different substrates. They filmed at ∼2000 Hz with a Chronos 1.4 camera and external illumination from a Westcott Skylux light. Photos used with permission from students. Photo credits A) C. E. Oufiero and B–D) K. Takeno.

The course resulted in four unique, independent research projects (Table 3) that were developed by the students with input from me on experimental design (Fig. 2). One group was inspired by the crayfish I initially had in the lab and researched how the presence of their chelae affect their escape performance. This was based on observations of variation in chelae presence among crayfish caught in the field. The second group was inspired by the water striders they observed in the creek while collecting crayfish and wanted to investigate their locomotor performance on different aquatic substrates. After researching previous studies, they learned about the structure and hydrophobicity of their setae, and they wanted to see if various aquatic mediums would result in different performances. The third group was inspired by a paper we discussed on hindlimb length affecting jump performance in froghoppers (Burrows and Sutton 2008) and wanted to determine if similar relationships were found in grasshoppers, crickets, and/or katydids after we caught several species in the field. The last group was inspired by all the invasive stink bugs and their known poor flight performance in homes in the fall. They focused on the effect of body mass on take-off velocity, which was discussed briefly in lectures. To emphasize, these were not prescribed by me, these were developed by the students, I simply helped them refine their experimental design and questions.

Keeping research questions a constant theme, discussing primary literature, evaluating early drafts of research questions and abstracts, and meeting with groups ensured that students did not get lost while trying to design their experiments. Furthermore, allowing students to fail and refine their experiments ensured they were on track. For example, one student wanted to work on dragonfly flight, but quickly decided against this when he couldn’t catch any in the field. The group that worked on jumping initially wanted to compare species, but once some species started to die, we refined their question to focus on the one species they still had. Lastly, because the students were evaluated on the process, they would not fail the course if the animals did not perform or their experiments failed.

Once groups troubleshot getting videos of their animals performing, they proceeded to film, digitize, and test their hypotheses, with all groups collecting complete datasets. Throughout the process students acquired several hard and soft skills. Hard skills included high-speed cinematography, data collection and management, using R to obtain performance data, making graphs, and analyze data, write a scientific paper, and present their results. Soft skills included working in groups, communication within their group, and time management. Not all groups worked as effectively, but all managed to complete their projects and assignments based on the given rubrics. For each group assignment, students had to evaluate their peers in the group (Supplementary Material). This ensured that the groups could distribute the work effectively and not have someone doing all or none of the work as it would be reflected in their grade for the group assignments.

The end result of the course was 12 students obtaining authentic research experiences. While it is always hoped a research project would lead to publishable results, the groups did not have to produce publishable results to do well in the course. Students were evaluated on the process of science not the outcomes. Many students felt bad if they did not get a result they expected, but that in itself is learning about the process of science. One of the group’s projects is currently being written up for potential publication, this was based on the novelty of the experiment, the quality of the data collected, and the results. This did not influence their grade. Three students from the course have also continued to conduct research in my own personal research lab, continuing with their research from the course (*N* = 1) or on other related projects being conducted in my lab (*N* = 2). A benefit of a model that is tangentially related to the professor’s research program is that if a project fails the professors research is not affected and the students still gain the experience. However, the other added benefit is that if there is a discovery that is interesting and worth pursing, it could be added to the research program and the students working on the project have the potential to continue working on it in BIOL 490 (research experience) or BIOL 491 (independent research). One goal of a CURE is to use the class time to train students in the techniques your lab uses, so the transition to conducting research in your personal lab is easier and they can become mentors to new students.

The videos students collected were highlighted as a video of the week on the course’s website (wp.towson.edu/off-lab) and videos students were collecting as well as their research movie trailers were shared on social media. This highlighted the importance of communicating research to a broader audience to the students. For example, one group’s video of stinkbug flight posted on YouTube was used in an online article about stink bugs (http://mentalfloss.com/article/561668/stink-bug-facts). The research movie trailers were shared on social media, and to the faculty, staff, and grad students in the department. During the poster day, each group had a sign-up sheet for visitors that came based on their videos, and bonus points were awarded to the group with the most visitors based on their research abstract movie trailer.

Another goal of this course and similar CUREs is for students to obtain ownership of their research and data they collect. This was evident in the qualitative portion of the course evaluations. Some of the qualitative results in response to the question, “what did you like about this course,” were

- I had the ability to make my own project.
- This was probably the most interesting course I have ever taken. It was a research based class which allowed me to be creative on what I was interested in.
- I liked the freedom of the research methods and project ideas.
- This course was perfect for anyone who needed research experience. It was great because we really started from the beginning with developing an idea, collected specimens, and finally producing research. I have never had a class as hands on as this one. This should be a requirement for all biomajors! It was a well put together course that has taught me so much.
- I liked that this course offered a more authentic research experience based on topics of our own interest and curiosity.

### Caveats about the course

While there were many positives to this course, there were also some challenges and areas where the course was not as effective. This included my use of peer evaluation, weather, computer literacy, and varying operating systems. First, since I had never taught a course that focused as much on group work, I decided I needed to have a mechanism in place for students to evaluate their peers to ensure even distribution of work. Using a standard form (Supplementary Material) I changed the points based on the assignment, with the peer evaluation counting for 40% of the total grade for group assignments. My short coming for the peer evaluation was how I had it submitted and grades entered into our online course websites on Balckboard (https://www.blackboard.com/index.html). I had each group submit one copy of their assignment into Balckboard, then each individual submit the peer evaluation. Therefore, when I graded the peer evaluation, students would see how the group did versus how they did, which does not make the peer evaluation anonymous. The peer evaluation also did not work in this manner for the group of two, as the other student knew exactly who graded them poorly. In future iterations, I will use the same, or similar, peer evaluation form, but I will likely change how I show grades on Blackboard so the group project and peer evaluation are not separate. The qualitative portion was helpful as I could see if there were problems that needed intervention.

Another challenge in a course that requires specimens collected from the field is weather. I had one contingency day build into the schedule, but I had to readjust the syllabus for the first 7 weeks because of rain (Supplementary Material). Because the class had a defined schedule, we could not go out any day the weather permitted. Therefore, instead of three field trips to two sites, we only had two to one site. At first, I was concerned with collecting animals, but luckily did some scouting beforehand at both sites to identify potential places to collect. I may add more collection sites in the future, but Oregon Ridge and our field station are close and amenable to us working there. Related to weather is keeping animals. The crayfish group had a massive die off over the first weekend because of one dying and poisoning the tank, which required them to make an additional trip out to the field with the grad student. Also, several species of grasshoppers died in the lab, likely because they were adults and close to the end of their life cycle.

An unexpected challenge throughout the course was computer literacy and varying operating systems. In previous courses where I have students use ImageJ, I have varied between having them use their own computers, and having them use university computers. I started in ImageJ and R in our computer lab, but then switched to their own personal computers so they could work at home. During one of our impromptu rained out field days we spent the entire Monday class period troubleshooting R and getting some initial kinematic code to work on personal computers (Fig. 1). I will likely build this day into future schedules as it turned out to be incredibly helpful for the students and me (Supplementary Material). With all the varying operating systems, it was tough for me to troubleshoot why programs weren’t working or loading. Having the ULA and grad student with some experience in R helped. There was also variation in students’ computer literacy, some took to using R very well, others did not. More of our courses at Towson are introducing R, so it is hoped this will be less of a problem in the future. But, having a day to troubleshoot R and kinematics was essential.

Lastly, the first time teaching this course, it was fairly unsettling not knowing what questions students would ask. There are a lot of unknowns at the start of a course where the animals, questions, and outcomes are up to the students, which can add stress to professors and students. It was unknown whether students could develop questions, if enough animals could be collected, and if animals would perform, but having grades based off the process ensures students will succeed. All the unknowns and issues that arise demonstrate the real process of conducting research. During this first iteration of the course, after questions were developed, animals were collected, and performances were recorded, the stress of the unknowns began to disappear. In general, I learned to have flexibility in the syllabus and schedule and to change things as needed (Supplementary Material). Sacrificing content throughout the semester allowed for the flexibility to have days to troubleshoot the research.

There are additional things I would change about the course in future iterations (Supplementary Material). First, I would spend more time on writing and have the students submit a draft of their final paper as a graded assignment. Having a draft submission would allow me to review their papers before they submit the final draft, provide more feedback on their writing and improve those skills, and potentially get papers closer to submission where applicable. I would also limit some of my lectures or break them up, such as muscle energetics and physiology. To ensure students are researching the primary literature more than the one or two papers they had to present on, I would also include an annotated bibliography as an assignment, with a presentation on one of the papers. To ensure the videos collected throughout the semester are consistent in their quality, I may have more checkpoint assignments for them. Lastly, I did not formally assess this course during the first iteration due to time and a lack of specifics I was interested in assessing. Instead, I decided to develop and offer the course a first time, and plan on formally assessing it during the next several iterations to obtain a quantitative view of how this organismal CURE is impacting students.

## Discussion

### How does this course differ from other courses?

There are several differences between this CURE, other CUREs, and traditional courses, as well as some similarities. My organismal form and function course differed from a similar course at UC Berkeley (Full et al. 2015), in that it focused solely on the use of high-speed cameras and animal movement, but was diverse in the questions and organisms that students worked on. Despite the difference between this CURE and Full et al.’s (2015), it is encouraging that more organismal CUREs are being developed (Table 2) to offer students research experiences in organismal biology. Many have moved to implementing research experiences or hypothesis testing within the lab portion of a traditional course over a couple of weeks, ending in a lab report (Auchincloss et al. 2014). The difference with the organismal form and function CURE is that content was sacrificed to provide time for the students to conduct research during class time. Traditionally, students take courses and conduct research outside of courses, sometimes receiving credit. The goal with this CURE was to provide time within the classroom period to conduct research. Therefore, I only covered a small portion of organismal form, function, and performance (Table 2), as the goal was not to instruct students on every topic, but provide the seeds to let their ideas grow. As the research questions were not up to me, and therefore unknown prior to the start of the semester, I chose topics we would likely encounter investigating invertebrate movement (muscle physiology, terrestrial locomotion, jumping, and flying). However, two groups worked on systems not related to the topics we covered in class. One group investigated water strider performance, which is a unique form of locomotion where the animals do not break the surface tension of the water (Hu et al. 2003). I did not cover this as it is a fairly specialized form of locomotion. They found the papers on how water striders achieve the ability to walk on water, which was unknown to me. Another group decided to work on crayfish escape responses. Even though some of my background is in fish swimming, I did not cover aquatic locomotion as I did not anticipate a group working on aquatic locomotion. I did provide some crayfish at the start that were purchased just to get students filming an animal, but once some students noticed the variation in presence of claws, they used this as their impetus for a research question. They researched the species that was collected and found papers describing the different types of escape responses.

A key to making research more inclusive is to provide dedicated course time for students to conduct research. In an area such as Baltimore where students may not have the resources, both time and supplies, to continue the research at home, the course provides that time. This provides the opportunity for students to develop their ideas, obtain the content necessary to conduct the research, research and present primary literature, analyze data, and even experience the messiness and failures of research (e.g., animals don’t always want to perform). Instead of collecting data in class, but leaving the analyses and research of prior literature and writing to outside of class, the course provided dedicated time during course time for these activities. This is crucial to make research opportunities more inclusive. Therefore, potentially more students can conduct research without any limitations. Teaching a course like this you have to be able to be adaptable and sacrifice the content to let the students make their own discoveries, an essential component of a CURE and scientific research. It is this discovery that provides them with ownership of their data and makes research more inclusive.

### How could others implement a similar course?

A goal of this course and paper is to inspire others to implement similar courses at other universities, potentially allowing for intercampus collaboration. Although this course was funded by the HHMI Inclusive Excellence Initiative, a lack of funding should not be a limitation. Most phones and action sports cameras (e.g., GoPro) have the capability to film at frame rates of 250 Hz, which would be sufficient for most animal performances we measured. I had a Fastec IL3 (Fastec Imaging, San Diego, CA) purchased off of research startup, as well as two Edgertronic SC1 cameras acquired from the HHMI grant (Sanstreak Corp., San Jose, CA), and a Chronos 1.4 camera acquired from a small internal grant (Kron Technologies, Inc., Burnaby, Canada). Both the Edgertronic and Chronos are providing high-speed capabilities at more affordable prices and were excellent for this course. Most students filmed at 500 Hz, but all cameras had the capability to film at higher frame rates with reduced resolution. High-speed cameras and associated sensors have also improved in their low light filming capabilities. The course had several high output LED lights (Naila Zila and Wescott Skylux) that were used for filming, one group solely relied on overhead lights without any loss of resolution, and one Edgertronic camera had the IR filter removed so performance could be captured with inexpensive CM Vision 850 nm LED lights (C&M Vision Technologies, Houston, TX). This setup was also good because it avoided the bright lights traditionally used to film at high-speeds, which can affect animal performance. While there are many options for tripods, I decided to get relatively inexpensive tripods as all of the cameras are not heavy (Magnus VT-300 Video Tripod with Fluid Head). To digitize and analyze videos we solely used open source software, including ImageJ and the MTrackJ plugin, Tracker (https://physlets.org/tracker/) to digitize video files from the Chronos cameras that were saved as .mov and not .avi files, and base R for kinematics and the lme4 function for statistical analyses (Bates et al. 2015). There is a large upfront cost to the course, but with lab fees the course should be able to run on a minimal budget to cover equipment repairs, replacement parts, animal housing enclosures, and money for field trips to collect.

The structure of this CURE focused on organismal form and function, which I specifically kept vague to allow flexibility. This framework could be implemented for any organism, including plants. Although other organisms may require different types of data collected, that is not high-speed videos, keeping the topic general allows others to teach this CURE. The course could also incorporate vertebrates and step the students through the process of writing an IACUC protocol, although this may not be feasible at all institutions and may take more time depending on the university or college. The course could be modified to focus on a specific group (e.g., beetles), a specific question (e.g., urban versus rural), or a specific performance (e.g., jumping). However, since I wanted students to have the freedom, I kept it fairly open. One aspect of teaching a CURE is the repeatability of science, if there are more implementations of organismal form and function CUREs a network could be developed to share and collaborate across institutions, offering the possibility of even broader research questions (e.g., comparisons across geographic regions).

## Conclusion

Transitioning to instructing students in authentic research experiences through a CURE is both rewarding and challenging. On the one hand, you get to train students in research in your area of expertise. Students may make discoveries, professors may get inspiration for new avenue of research, and professors get to interact with students on a much more personal level then in a large lecture. On the other hand, you may not know what the students will ask. Some students may struggle with developing a question and the non-traditional framework of the class. Projects may fail, which can be frustrating to students. Animals may not cooperate. However, these are all part of the authentic research experiences. Evaluating students on the process of science, and not the results, helps alleviate some frustrations, while still instructing students in the scientific process. A key to teaching this type of course is allowing flexibility in the syllabus and having contingency plans. I decided not to include some assignments I initially intended to, but switched them to better represent where the class was. In the end, I think the course was a success for me and the students. They obtain research experience, several have continued working on research, and I was able to instruct students in organismal form and function.

## Supplementary Material

obz021_Supplementary_Data
