# Supplementary material for: The Organismal Form and Function Lab-Course: A New CURE for a Lack of Authentic Research Experiences in Organismal Biology
Source: Integr Org Biol. 2019 Aug 23;1(1):obz021. doi: 10.1093/iob/obz021 (PMC7671133; doi:10.1093/iob/obz021)
Supplement: obz021_Supplementary_Data [file obz021_supplementary_data.docx]

**Supplementary material**

Rubric to evaluate high-speed videos for kinematic/performance analysis.

|  | **Excellent (1)** | **Good (0.5)** | **Unacceptable (0)** | **Score** |
| --- | --- | --- | --- | --- |
| **Focus** | Animal is in the depth of field with sharp focus, no blurred edges. Entire video remains in focus. | Animal is in the depth of field with mostly sharp focus, edges are blurred a bit. Focus is lost throughout video. | Animal is not in focus, edges are blurred. |  |
| **Blur** | There is no blur from frame to frame. Shutter speed and frame rate are fast enough so each frame is sharp, allowing the identification of edges | There is minimal blur from frame to frame. The shutter speed and or frame rate could be adjusted better to minimize frame to frame blur. | There is too much blur from frame to frame making analyses difficult or impossible. The shutter speed and or frame rate should be adjusted to minimize blur. |  |
| **Light** | The light is bright enough, but not too bright for digitizing throughout the video | The video is a bit washed out or dark, but points could still be digitized. | The lighting is too bright or dark, making the video inappropriate for analysis. |  |
| **Composition-**  **2 dimensional** | The animal remains in a position to capture the 2D behavior throughout video, it does not go in the 3^rd^ dimension, which would alter results | The animal is mostly in a 2D position to capture the behavior, but may go into the 3^rd^ dimensions a bit. Video could still be used for digitizing. | The animal goes in to the 3^rd^ dimension and is not the right orientation which would distort any resulting kinematics. |  |
| **Performance/behavior** | The video captures the entire performance or behavior of interest | The video captures most of the behavior/performance, but some aspects might be missing. | The video does not fully capture the behavior/performance and is not useful for analysis. |  |

Rubric to assess student movie trailer research abstracts. Example trailers can be found at <https://www.youtube.com/channel/UCHwfwTS-hNyhZ9Xxi0BgVCA/featured>

|  | **Exemplary (6)** | **Acceptable (4-5)** | **Developing (2-3)** | **Unacceptable (0-1)** | **Score** |
| --- | --- | --- | --- | --- | --- |
| **Research content** | Video clearly presents the research topic, including the study animal, the questions/hypotheses and the methods. What is being tested and why is clearly presented. | Video presents the research topic, and mostly includes the study animal, the questions/hypotheses and the methods. What is being tested and why is presented. | Video presents the research topic, but several components are missing (e.g., study animal, the questions/hypotheses or the methods). What is being tested and why is not clearly presented. | Video does not clearly present the research topic, with several components missing (e.g., study animal, the questions/hypotheses or the methods). What is being tested and why is not presented. |  |
| **Video clarity** | The video is in focus, has good lighting, and demonstrates the research videos that are being used to address the questions. The flow and editing of the video are appropriate to keep attention. | The video is mostly in focus, has decent lighting, and demonstrates some of the research videos that are being used to address the questions. The flow and editing of the video could be improved. | The video is mostly not in focus, has poor lighting, and does not demonstrate the research videos that are being used to address the questions. The flow and editing of the video are not appropriate to keep attention. | The video is not focus, has very poor lighting, and does not demonstrate any of the research videos that are being used to address the questions. The flow and editing of the video are not appropriate to keep attention. |  |
| **Questions/ hypothesis** | The questions and hypotheses are clearly stated in the video. The audience knows exactly what is being addressed and why it is being researched. | The questions and hypotheses are stated in the video. The audience knows what is being addressed and why it is being researched. | The questions and hypotheses are not clearly stated in the video. The audience may not know exactly what is being addressed and why it is being researched. | The questions and hypotheses are not stated in the video. The audience would not be able to determine what is being addressed and why it is being researched. |  |
| **Methods** | The methods are clearly presented in the video to a point where the audience can understand how the researchers will address their research questions. It is very clear how the videos they’re obtaining will ask the questions they’re asking. | The methods are presented in the video to a point where the audience can mostly understand how the researchers will address their research questions. It is clear how the videos they’re obtaining will ask the questions they’re asking. | Some of the methods are presented in the video to a point where the audience may not understand how the researchers will address their research questions. It is not clear how the videos they’re obtaining will ask the questions they’re asking. | The methods are not clearly presented in the video to a point where the audience will not be able to understand how the researchers will address their research questions. It is not clear how the videos they’re obtaining will ask the questions they’re asking. |  |
| **Creativity** | The video is engaging, it grabs the audience’s attention, but also emphasizes the research. The video does an excellent job of connecting the research to the audience in a unique manner, with a balance between creative content and scientific content | The video is engaging, it may grab the audience’s attention, but the emphasis on research is not as strong. The video does a decent job of connecting the research to the audience in a unique manner, with a decent balance between creative content and scientific content | The video is lacking in engaging the audience, it may not grab their attention. The emphasis on research is lacking. The video does a poor job of connecting the research to the audience in a unique manner. There is a lack of balance between creative content and scientific content | The video is not engaging, and may not grab the audience’s attention. The emphasis on research is lacking. There is no connection of the research to the audience in a unique manner. There is a lack of balance between creative content and scientific content |  |

Sample peer evaluation form:

**Peer Evaluation Form for Group Work**

Your name ____________________________________________________

Write the name of each of your group members in a separate column. For each person, indicate the extent to which you agree with the statement on the left, using a scale of 1-4 (1=strongly disagree; 2=disagree; 3=agree; 4=strongly agree). Total the numbers in each column.

| Evaluation Criteria | Group member: | Group member: | Group member: | Group member: |
| --- | --- | --- | --- | --- |
| Attends group meetings regularly and arrives on time. |  |  |  |  |
| Contributes meaningfully to group discussions and demonstrates a cooperative and supportive attitude. |  |  |  |  |
| Completes group assignments on time. |  |  |  |  |
| Prepares work in a quality manner. |  |  |  |  |
| Contributes significantly to the success of the project. |  |  |  |  |
| TOTALS |  |  |  |  |

Feedback on team dynamics:

1. How effectively did your group work?
2. Were the behaviors of any of your team members particularly valuable or detrimental to the team? Explain.
3. What did you learn about working in a group from this project that you will carry into your next group experience?

Adapted from a peer evaluation form developed at Johns Hopkins University (October, 2006)

| Week | Day | Topic | Reading | Assignment |
| --- | --- | --- | --- | --- |
| 1 | 8/27  8/29 | Introduction: class, camera setup  Form, function and performance | Irschick and Higham Ch. 1 | **Due at end of lab**: video test (5 pts)  Discuss reading |
| 2 | **9/3** | **Labor Day NO CLASS** |  |  |
|  | 9/5 | What affects performance?  (paper discussion) | Irschick and Higham Ch. 2 | **DUE 9/5**: Paper summary (10 pts)  Paper: Winchell et al. 2018 |
| 3 | 9/10  9/12 | Introduction: kinematic and performance analyses  Muscle Physiology and Energetics | Biewener and Patek Ch. 2 | **DUE 9/10**: Two observations from nature (5 pts.) |
| 4 | 9/17 | Collection field trip (Oregon Ridge) |  | **DUE 9/17**: Digitizing test (10 pts.) |
|  | 9/19 | Terrestrial locomotion | Biewener and Patek Ch. 4 | Paper discussion: Full & Tullis 1990 |
| 5 | 9/24  9/26 | Collection Field trip (TU Field Station)  Jumping, climbing, clinging | Biewener and Patek Ch. 7 | **DUE 9/24**: Kinematic test (10 pts.)  Paper discussion: |
| 6 | 10/1  10/3 | Collection field trip/animal training  (Oregon Ridge)  Flying | Biewener and Patek Ch. 6 | **DUE 10/1**: Initial hypothesis/question draft (5 pts.)  Paper discussion: |
| 7 | 10/8 | Lab work |  | **DUE 10/8**: Video, digitized point, kinematic assignment (40 pts) **group** |
|  | **10/10** | **Midterm** |  |  |
| 8 | 10/15  10/17 | Lab work  Lab meeting | **TBD** | **DUE 10/17**: Initial **group** presentation of research (50 pts.) |
| 9 | 10/22  10/24 | Lab work  Data analysis and paper discussion | **TBD** | **DUE 10/22**: Paper summary (20 pts.)  **DUE 10/24**: 3 student paper presentations (20 points) |
| 10 | 10/29  10/31 | Lab work  Lab meeting and paper discussion | **TBD** | **DUE 10/31**: 3 student’s paper presentations (20 pts.) |
| 11 | 11/5 | Lab work |  |  |
|  | 11/7 | Lab meeting and paper discussions | **TBD** | **DUE 11/7**: 3 student’s paper presentations (20 pts.) |
| 12 | 11/12  11/14 | Lab work  Data presentation |  | **DUE 11/14**: **Group** Teaser trailer (50 pts.) |
| 13 | 11/19 | Lab work |  |  |
|  | **11/21** | **Thanksgiving NO CLASS** |  |  |
| 14 | 11/26  11/28 | Lab Work  Lab meeting and paper discussion |  | **DUE 11/28**: 3 student’s paper presentations (20 pts.) |
| 15 | 12/3 | Lab work & poster prep |  | Department of Biology **Group** poster presentation (50 points) |
|  | 12/5 | Lab meeting |  |  |
| 16 | 12/10  12/14 | Final presentation due |  | **DUE 12/10**: **Group** final presentation (50 points)  **DUE 5 pm 12/14: Group** final paper due (100 points) |

**Assignment Schedule**

| **Week** | **Assignment** | **Points** | **Due** | **Individual or Group** |
| --- | --- | --- | --- | --- |
| **1** | Video test | 5 | 8/27 end of class | Individual |
| **2** | Paper summary | 10 | 9/5/18 3pm | Individual |
| **3** | Field observations | 5 | 9/10/18 1pm | Individual |
| **4** | Digitizing test | 10 | 9/17/18 1pm | Individual |
| **5** | Kinematic test | 10 | 9/24/18 1pm | Individual |
| **6** | Initial hypotheses/questions | 5 | 10/1/18 1pm | Individual |
| **7** | Video, digitized, kinematic | 40 | 10/8/18 1pm | Group |
|  | **Midterm** | **75** | **10/10/18** | **Individual** |
| **8** | Presentation of project | 50 | 10/17/18 3 pm | Group |
| **9** | Paper summary  **3** student paper presentations | 20  20 | 10/22/18 1pm  10/24/18 3 pm | Individual  Individual |
| **10** | **3** student paper presentations | 20 | 10/31/18 3pm | Individual |
| **11** | **3** student paper presentations | 20 | 11/7/18 3pm | Individual |
| **12** | Teaser Trailer | 50 | 11/14/18 3pm | Group |
| **13** |  |  |  |  |
| **14** | **3** student paper presentations | 20 | 11/28/18 3pm | Individual |
| **15** | Dept. poster presentation | 50 | 12/7/18  Friday 11-1:30Paper | Group |
| **16** | Final presentation  Final Paper | 50  100 | 12/10/18 1pm  12/14/18 5pm | Group  Group |

| Week | Day | Topic | Reading | Assignment |
| --- | --- | --- | --- | --- |
| 1 | 8/27  8/29 | Introduction: class, camera setup  Form, function and performance | Irschick and Higham Ch. 1 | **Due at end of lab**: video test (5 pts)  Discuss reading |
| 2 | **9/3** | **Labor Day NO CLASS** |  |  |
|  | 9/5 | What affects performance?  (paper discussion) | Irschick and Higham Ch. 2 | **DUE 9/5**: Paper summary (10 pts)  Paper: Winchell et al. 2018 |
| 3 | 9/10  9/12 | Introduction: kinematic and performance analyses, practice filming  Muscle Physiology and Energetics | Biewener and Patek Ch. 2 | **DUE 9/10**: Two observations from nature (5 pts.) |
| 4 | 9/17 | Practice filming, collecting in arboretum |  | **DUE 9/17**: Digitizing test (10 pts.) |
|  | 9/19 | Terrestrial locomotion | Biewener and Patek Ch. 4 | Paper discussion: Full & Tullis 1990 |
| 5 | 9/24  9/26 | Troubleshoot R for kinematics, practice filming  Jumping, climbing, clinging | Biewener and Patek Ch. 7 | **DUE 9/24**: Kinematic test (10 pts.)  Paper discussion: Burrows and Sutton 2008 |
| 6 | 10/1  10/3 | Collection field trip Oregon Ridge  Flying | Biewener and Patek Ch. 6 | **DUE 10/1**: Initial hypothesis/question draft (5 pts.)  Paper discussion: Crall et al. 2015 |
| 7 | 10/8 | Collection Trip Oregon Ridge |  |  |
|  | **10/10** | **Midterm** |  | **DUE 10/12/18:** research proposal abstract (30 points) |
| 8 | 10/15  10/17 | Lab work, meet with groups to work on experimental design  Lab work | **TBD** |  |
| 9 | 10/22  10/24 | Lab work  Presentation and paper discussion | **TBD** | **DUE 10/22/18**: Video, digitized point, kinematic assignment (40 pts) **group**  **DUE 10/24**: 3 student paper presentations (20 points) |
| 10 | 10/29  10/31 | Lab work  Lab meeting and paper discussion | **TBD** | **DUE 10/31**: 3 student’s paper presentations (20 pts.)  **DUE 10/31**: Initial **group** presentation of research (50 pts.) |
| 11 | 11/5 | Lab work |  |  |
|  | 11/7 | Lab meeting and paper discussions | **TBD** | **DUE 11/7**: 3 student’s paper presentations (20 pts.) |
| 12 | 11/12  11/14 | Lab work  Data analysis & Data presentation |  |  |
| 13 | 11/19 | Lab work |  | **DUE 11/19**: **Group** Teaser trailer (50 pts.) |
|  | **11/21** | **Thanksgiving NO CLASS** |  |  |
| 14 | 11/26  11/28 | Lab Work  Lab meeting and paper discussion |  | **DUE 11/28**: 3 student’s paper presentations (20 pts.) |
| 15 | 12/3  12/5 | Lab work & poster prep  Lab meeting |  | Department of Biology **Group** poster presentation (50 points) 12/7/18 |
| 16 | 12/10  12/14 | Final presentation due |  | **DUE 12/10**: **Group** final presentation (50 points)  **DUE 5 pm 12/14: Group** final paper due (100 points) |

**Assignment Schedule**

| **Week** | **Assignment** | **Points** | **Due** | **Individual or Group** |
| --- | --- | --- | --- | --- |
| **1** | Video test | 5 | 8/27 end of class | Individual |
| **2** | Paper summary | 10 | 9/5/18 3pm | Individual |
| **3** | Field observations | 5 | 9/10/18 1pm | Individual |
| **4** | Digitizing test | 10 | 9/17/18 1pm | Individual |
| **5** | Kinematic test | 10 | 9/24/18 1pm | Individual |
| **6** | Initial hypotheses/questions | 5 | 10/1/18 1pm | Individual |
| **7** |  |  |  |  |
|  | **Midterm** | **75** | **10/10/18** | **Individual** |
| **8** | Revised proposal abstract | 30 | 10/12/18 1 pm | Group |
| **9** | Video, digitized, kinematic  **3** student paper presentations | 40  20 | 10/22/18 1pm  10/24/18 3 pm | Group  Individual |
| **10** | Presentation of project  **3** student paper presentations | 50  20 | 10/31/18  10/31/18 3pm | Group  Individual |
| **11** | **3** student paper presentations | 20 | 11/7/18 3pm | Individual |
| **12** | Teaser Trailer | 50 | 11/19/18 3pm | Group |
| **13** |  |  |  |  |
| **14** | **3** student paper presentations | 20 | 11/28/18 3pm | Individual |
| **15** | Dept. poster presentation | 70 | 12/7/18  Friday 11-1:30Paper | Group |
| **16** | Final Paper | 100 | 12/14/18 5pm | Group |

| Week | Day | Topic | Reading | Assignment |
| --- | --- | --- | --- | --- |
| 1 | 8/27  8/29 | Introduction: class, camera setup  Form, function and performance | Irschick and Higham Ch. 1 | **Due at end of lab**: video test (5 pts)  Discuss reading |
| 2 | **9/3** | **Labor Day NO CLASS** |  |  |
|  | 9/5 | What affects performance?  (paper discussion) | Irschick and Higham Ch. 2 | **DUE 9/5**: Paper summary (10 pts)  Paper: Winchell et al. 2018 |
| 3 | 9/10  9/12 | Introduction: kinematic and performance analyses, practice filming  Muscle Physiology and Energetics | Biewener and Patek Ch. 2 | **DUE 9/10**: Two observations from nature (5 pts.) |
| 4 | 9/17 | Practice filming, collecting in arboretum |  | **DUE 9/17**: Digitizing test (10 pts.) |
|  | 9/19 | Muscle continued, Terrestrial locomotion | Biewener and Patek Ch. 4 | Paper discussion: Full & Tullis 1990 |
| 5 | 9/24  9/26 | Troubleshoot R for kinematics, practice filming  Jumping, climbing, clinging | Biewener and Patek Ch. 7 | **DUE 9/24**: Kinematic test (10 pts.)  Paper discussion: Burrows and Sutton 2008 |
| 6 | 10/1  10/3 | Collection field trip Oregon Ridge  Flying | Biewener and Patek Ch. 6 | **DUE 10/1**: Initial hypothesis/question draft (5 pts.)  Paper discussion: Crall et al. 2015 |
| 7 | 10/8 | Collection Trip Oregon Ridge |  |  |
|  | **10/10** | **Midterm** |  | **DUE 10/12:** research proposal abstract (30 points) |
| 8 | 10/15  10/17 | Lab work, meet with groups to work on experimental design  Lab work | **TBD** |  |
| 9 | 10/22  10/24 | Lab work  Presentation and paper discussion | **TBD** | **DUE 10/22**: Video, digitized point, kinematic assignment (40 pts) **group**  **DUE 10/24**: 3 student paper presentations (20 points) |
| 10 | 10/29  10/31 | Lab work  Lab meeting and paper discussion | **TBD** | **DUE 10/31**: 3 student’s paper presentations (20 pts.)  **DUE 10/31**: Initial **group** presentation of research (50 pts.) |
| 11 | 11/5 | Lab work |  |  |
|  | 11/7 | Lab meeting and paper discussions | **TBD** | **DUE 11/7**: 3 student’s paper presentations (20 pts.) |
| 12 | 11/12  11/14 | Lab work  Data analysis & Data presentation |  |  |
| 13 | 11/19 | Lab work |  | **DUE 11/19**: **Group** Teaser trailer (50 pts.) |
|  | **11/21** | **Thanksgiving NO CLASS** |  |  |
| 14 | 11/26  11/28 | Lab Work  Lab meeting and paper discussion |  | **DUE 11/26** Draft of final paper (30 points)  **DUE 11/28**: 3 student’s paper presentations (20 pts.) |
| 15 | 12/3  12/5 | Lab work & poster prep  Lab meeting |  | Department of Biology **Group** poster presentation (50 points) 12/7/18 |
| 16 | 12/10  12/14 |  |  | **DUE 12/10**: **Individual** annotated bibliography (50 points)  **DUE 5 pm 12/14: Group** final paper due (100 points) |

**Assignment Schedule**

| **Week** | **Assignment** | **Points** | **Due** | **Individual or Group** |
| --- | --- | --- | --- | --- |
| **1** | Video test | 5 | 8/27 end of class | Individual |
| **2** | Paper summary | 10 | 9/5 3pm | Individual |
| **3** | Field observations | 5 | 9/10 1pm | Individual |
| **4** | Digitizing test | 10 | 9/17 1pm | Individual |
| **5** | Kinematic test | 10 | 9/24 1pm | Individual |
| **6** | Initial hypotheses/questions | 5 | 10/1 1pm | Individual |
| **7** |  |  |  |  |
|  | **Midterm** | **75** | **10/10** | **Individual** |
| **8** | Revised proposal abstract | 30 | 10/12 1 pm | Group |
| **9** | Video, digitized, kinematic  **3** student paper presentations | 40  20 | 10/22 1pm  10/24 3 pm | Group  Individual |
| **10** | Presentation of project  **3** student paper presentations | 50  20 | 10/31  10/31 3pm | Group  Individual |
| **11** | **3** student paper presentations | 20 | 11/7 3pm | Individual |
| **12** | Teaser Trailer | 50 | 11/19 3pm | Group |
| **13** |  |  |  |  |
| **14** | Final Paper Draft  **3** student paper presentations | 30  20 | 11/26 5pm  11/28 3pm | Group  Individual |
| **15** | Dept. poster presentation | 70 | 12/7  Friday 11-1:30Paper | Group |
| **16** | Annotated Bibliography  Final Paper | 50  100 | 12/10 5pm  12/14 5pm | Individual  Group |
